# Supplementary figures and images for: Plasma metabolic profile reveals signatures of maternal health during gestational hypertension and preeclampsia without and with severe features
Source: PLoS One. 2024 Nov 26;19(11):e0314053. doi: 10.1371/journal.pone.0314053 (PMC11594399; doi:10.1371/journal.pone.0314053)

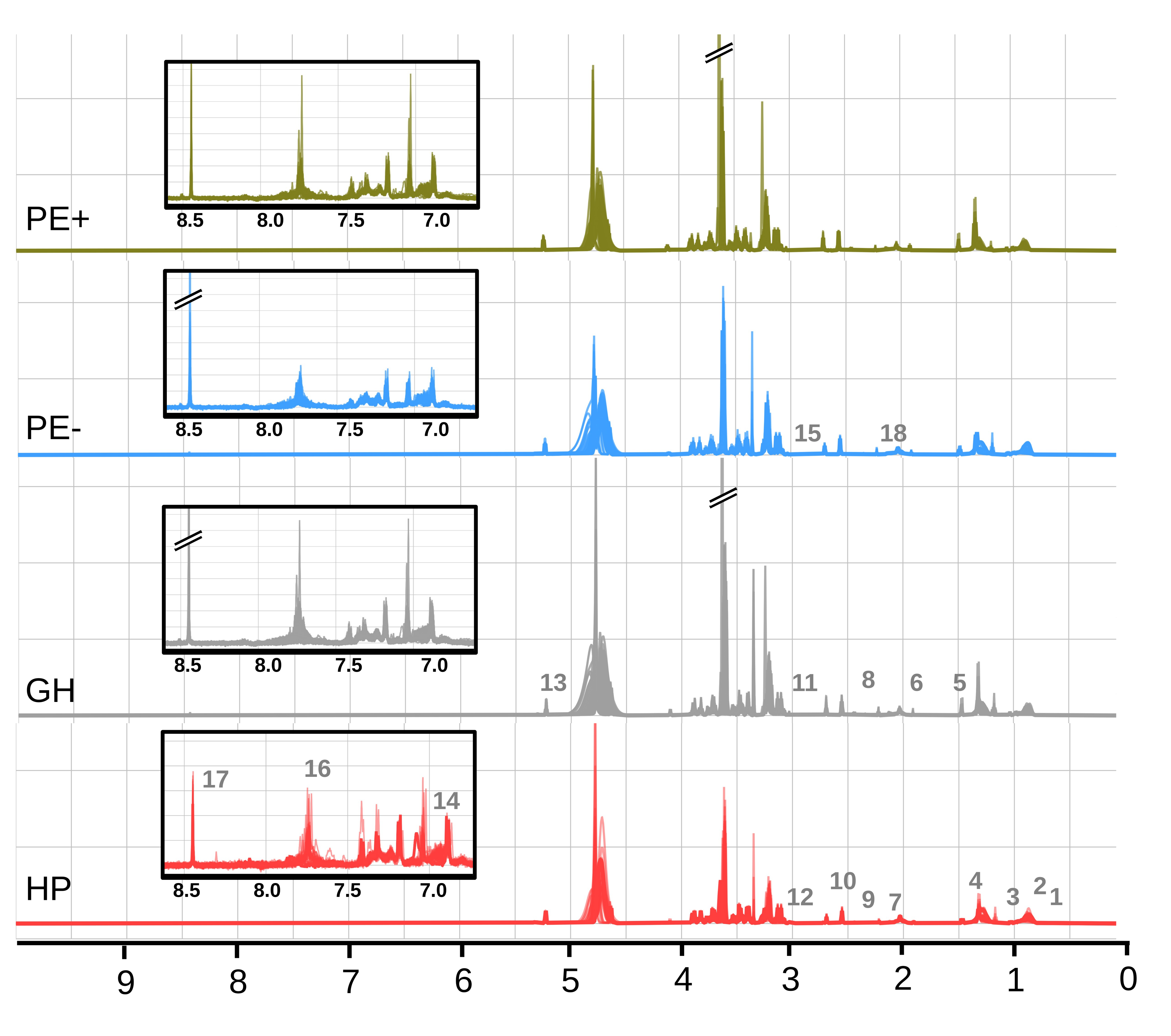

Supplement: S1 Fig — The spectra were acquired on the Bruker AVANCE III 600 MHz at 25°C and obtained by applying a 1H-NMR pulse sequence with a T2 filter (cpmgpr1d). 1, leucine; 2, isoleucine; 3, valine; 4, lactate; 5, alanine; 6, acetate; 7, acetone; 8, 3-hydroxybutyrate; 9, glutamine; 10, citrate; 11, creatine; 12, creatinine; 13, glucose; 14, tyrosine; 15, N,N-dimethylglycine; 16, histidine; 17, formate; 18, N-acetylglycoproteins. (TIF) [file pone.0314053.s001.tif]

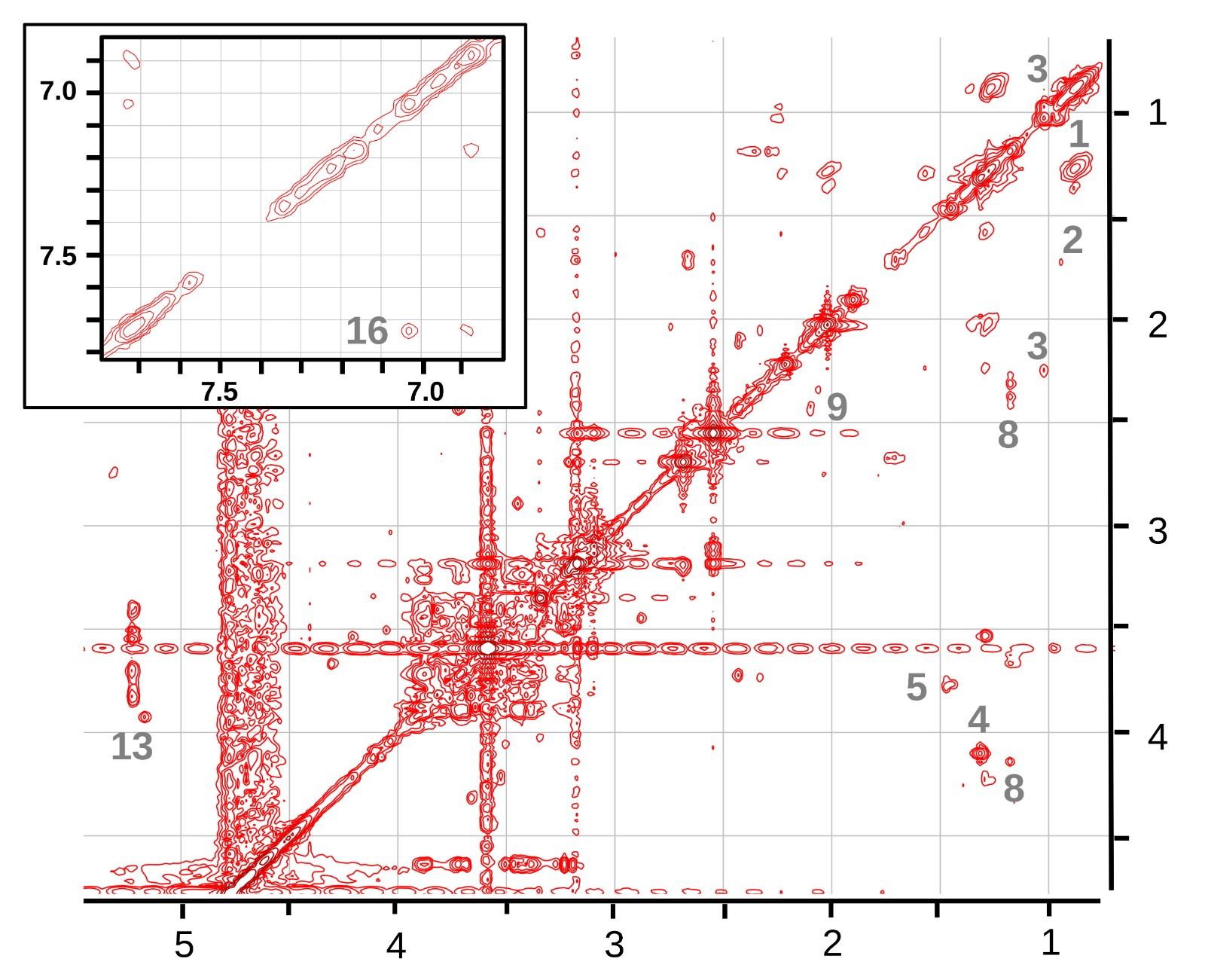

Supplement: S2 Fig — 1, leucine; 2, isoleucine; 3, valine; 4, lactate; 5, alanine; 6, acetate; 7, acetone; 8, 3-hydroxybutyrate; 9, glutamine; 10, citrate; 11, creatine; 12, creatinine; 13, glucose; 14, tyrosine; 15, N,N-dimethylglycine; 16, histidine; 17, formate; 18, N-acetylglycoproteins. (TIF) [file pone.0314053.s002.tif]

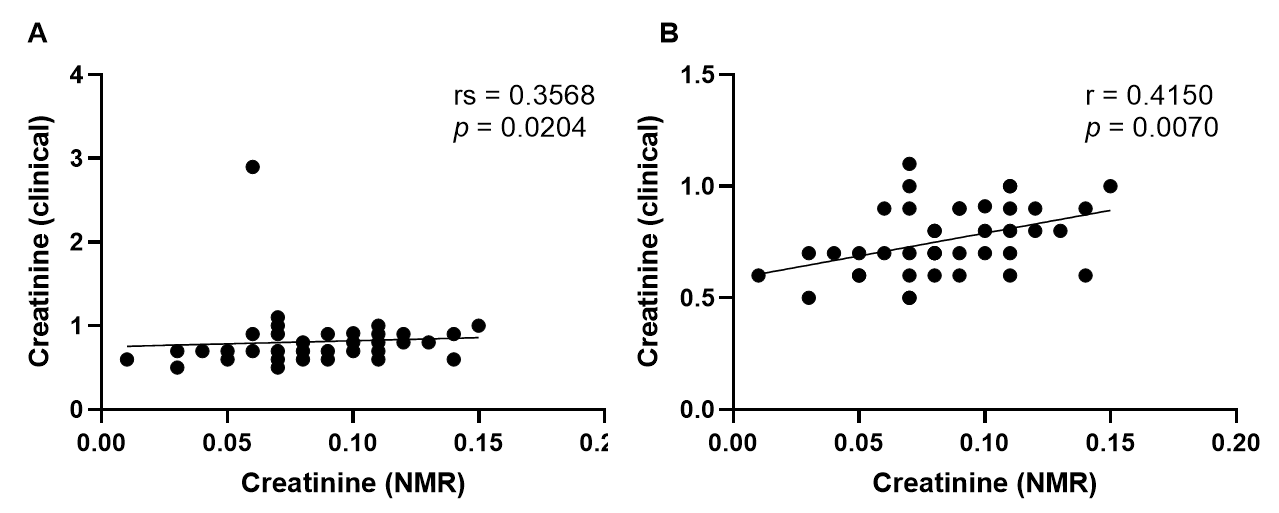

Supplement: S3 Fig — The rs value indicates the Spearman correlation coefficient, whereas the r value indicates the Pearson correlation coefficient. (TIF) [file pone.0314053.s003.tif]

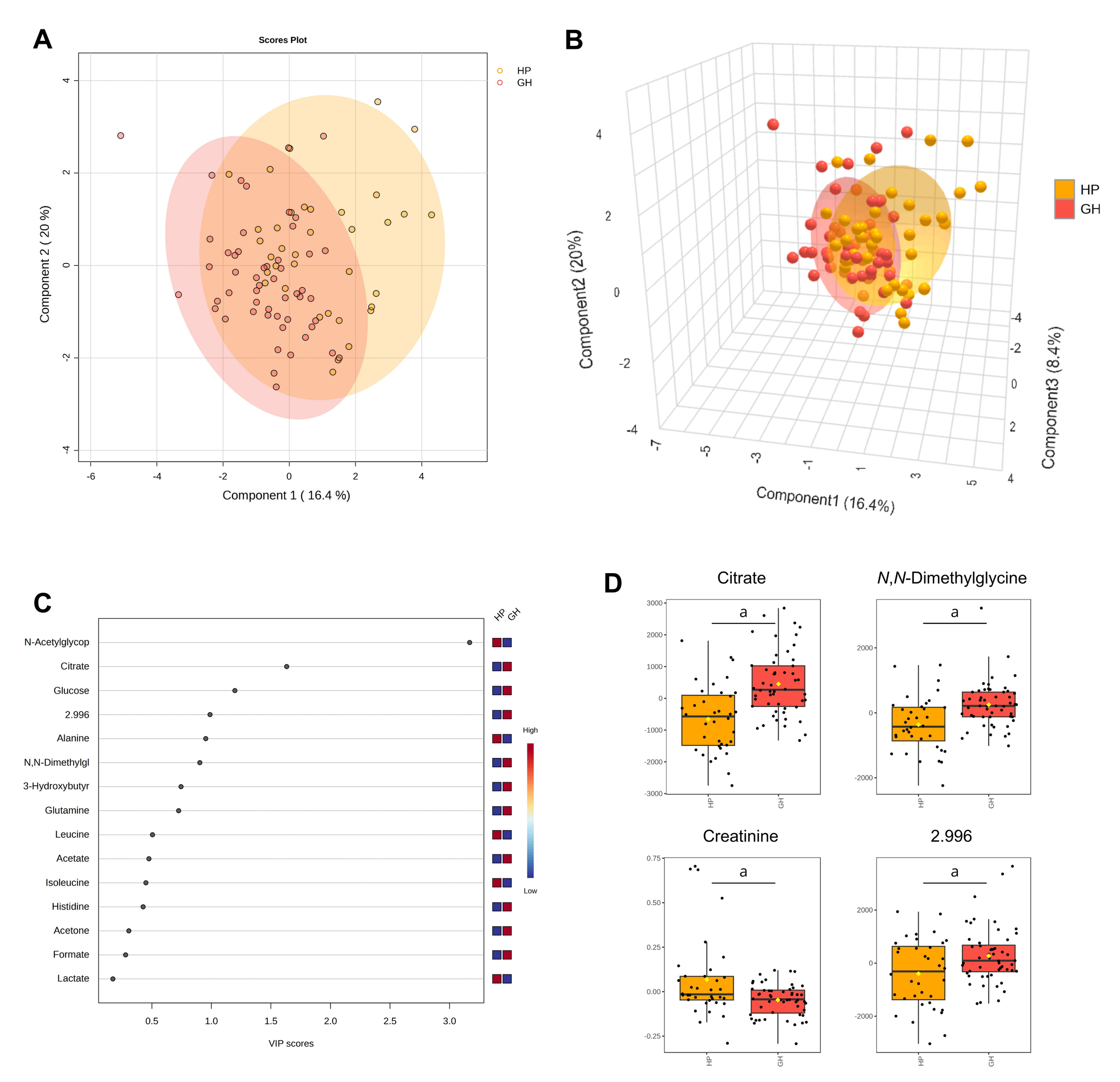

Supplement: S4 Fig — (A) 2-dimensional and (B) 3-dimensional score plots driven by sPLS-DA of the two experimental groups. (C) Variable importance in projection (VIP) scores spotlight the 15 most significant metabolites for group differentiation by PLS-DA. (D) Box plots showcasing variations in four metabolites with significant differences between HP and GH groups identified in the 1H-NMR spectra: citrate, N,N-dimethylglycine, creatinine, and 2.996. Data are shown as normalized plasma metabolite concentrations and statistically analyzed by Student’s t-test. p < 0.05 (a) were considered significant. HP (yellow) and GH (orange). (TIF) [file pone.0314053.s004.tif]

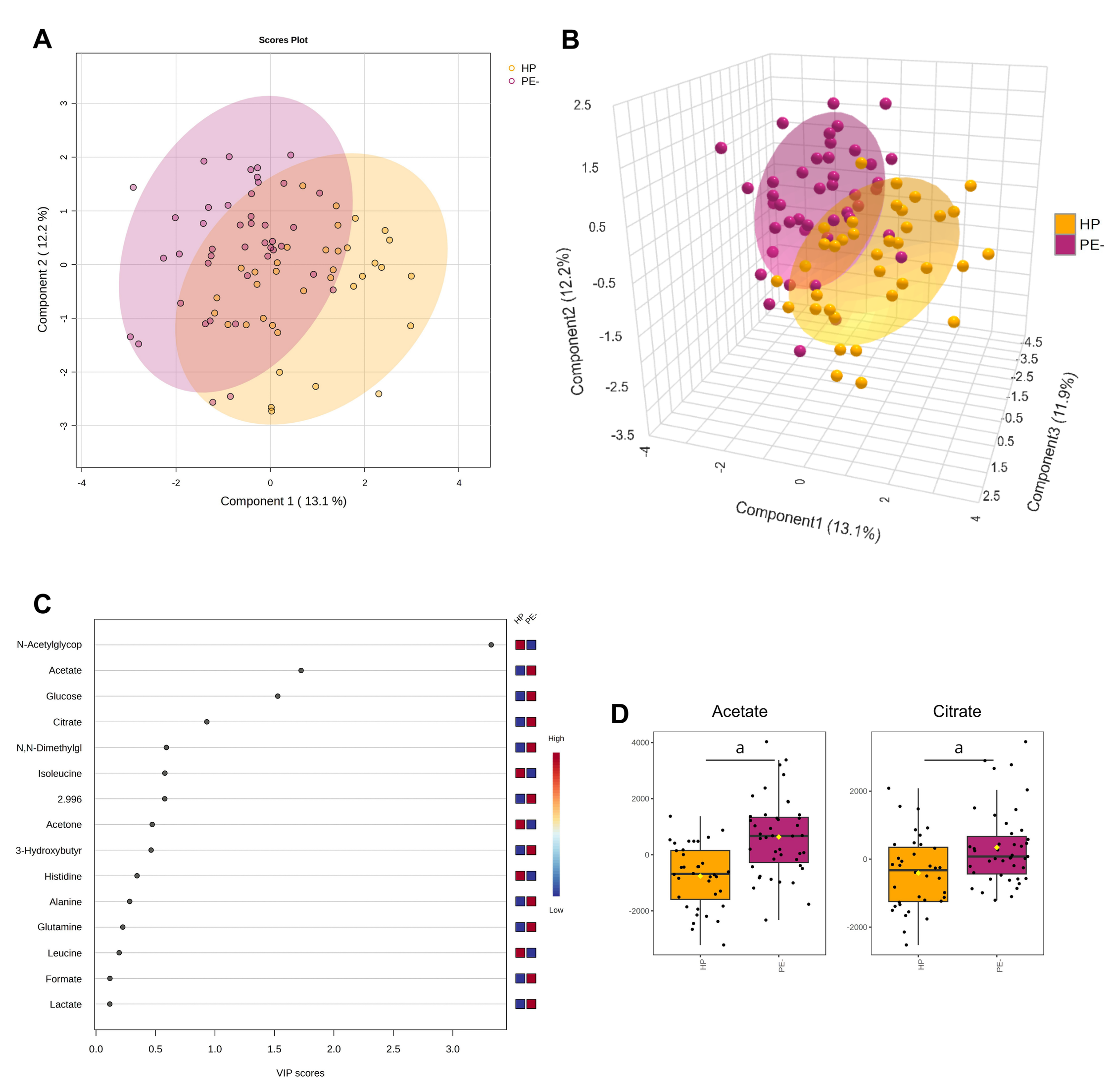

Supplement: S5 Fig — (A) 2-dimensional and (B) 3-dimensional score plots driven by sPLS-DA of the two experimental groups. (C) Variable importance in projection (VIP) scores spotlights the 15 most significant metabolites for group differentiation by PLS-DA. (D) Box plots showcasing variations in two metabolites with significant differences between HP and PE–groups identified in the 1H-NMR spectra: acetate and citrate. Data are shown as normalized plasma metabolite concentrations and statistically analyzed by Student’s t-test. p < 0.05 (a) were considered significant. HP (yellow) and PE–(pink). (TIF) [file pone.0314053.s005.tif]

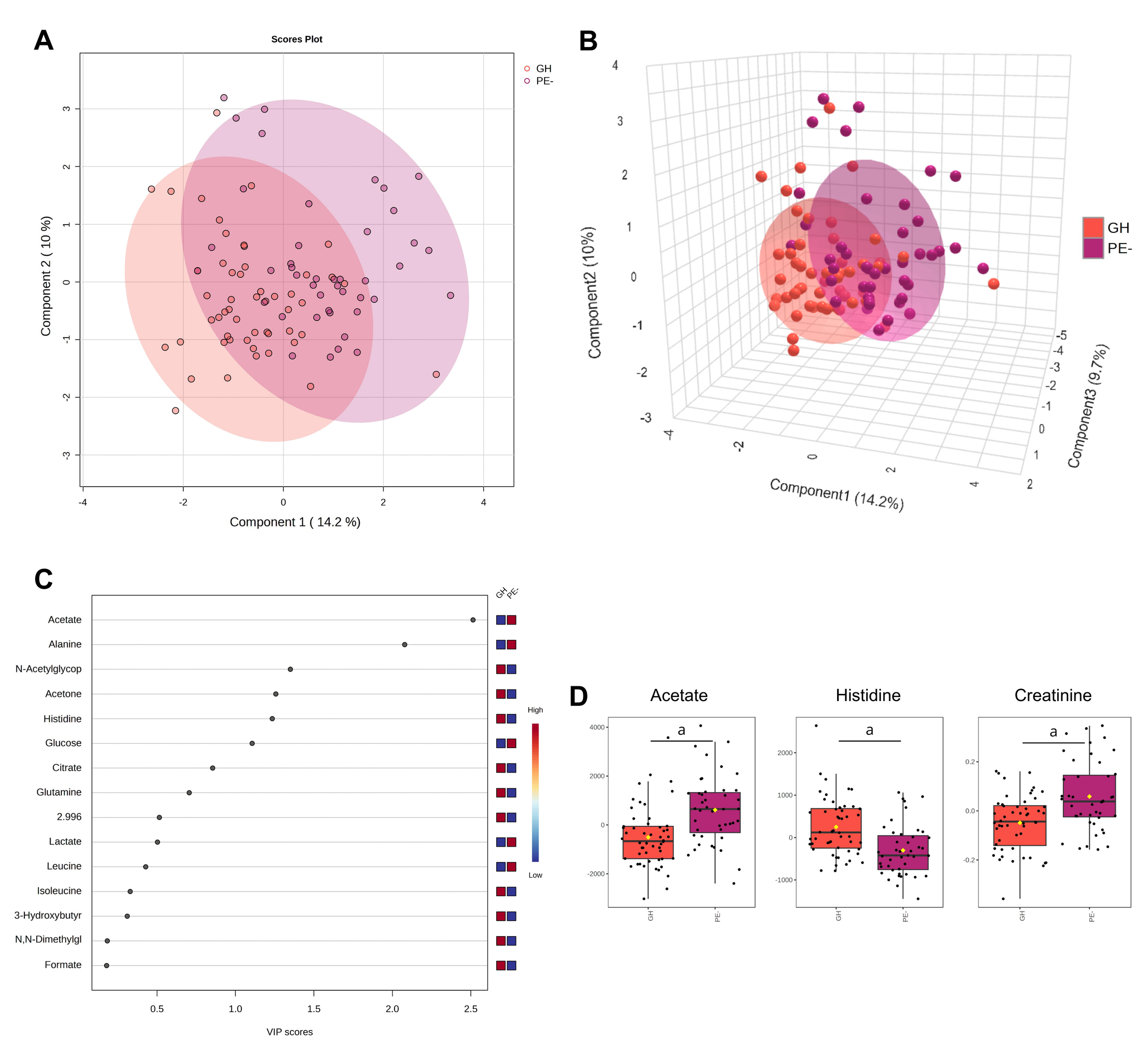

Supplement: S6 Fig — (A) 2-dimensional and (B) 3-dimensional score plots driven by sPLS-DA of the two experimental groups. (C) Variable importance in projection (VIP) scores spotlight the 15 most significant metabolites for group differentiation by PLS-DA. (D) Box plots showcasing variations in three significant metabolites between GH and PE–groups as identified in the 1H-NMR spectra: acetate, histidine, and creatinine. Data are shown as normalized plasma metabolite concentrations and statistically analyzed by Student’s t-test. p < 0.05 (a) were considered significant. GH (orange) and PE–(pink). (TIF) [file pone.0314053.s006.tif]

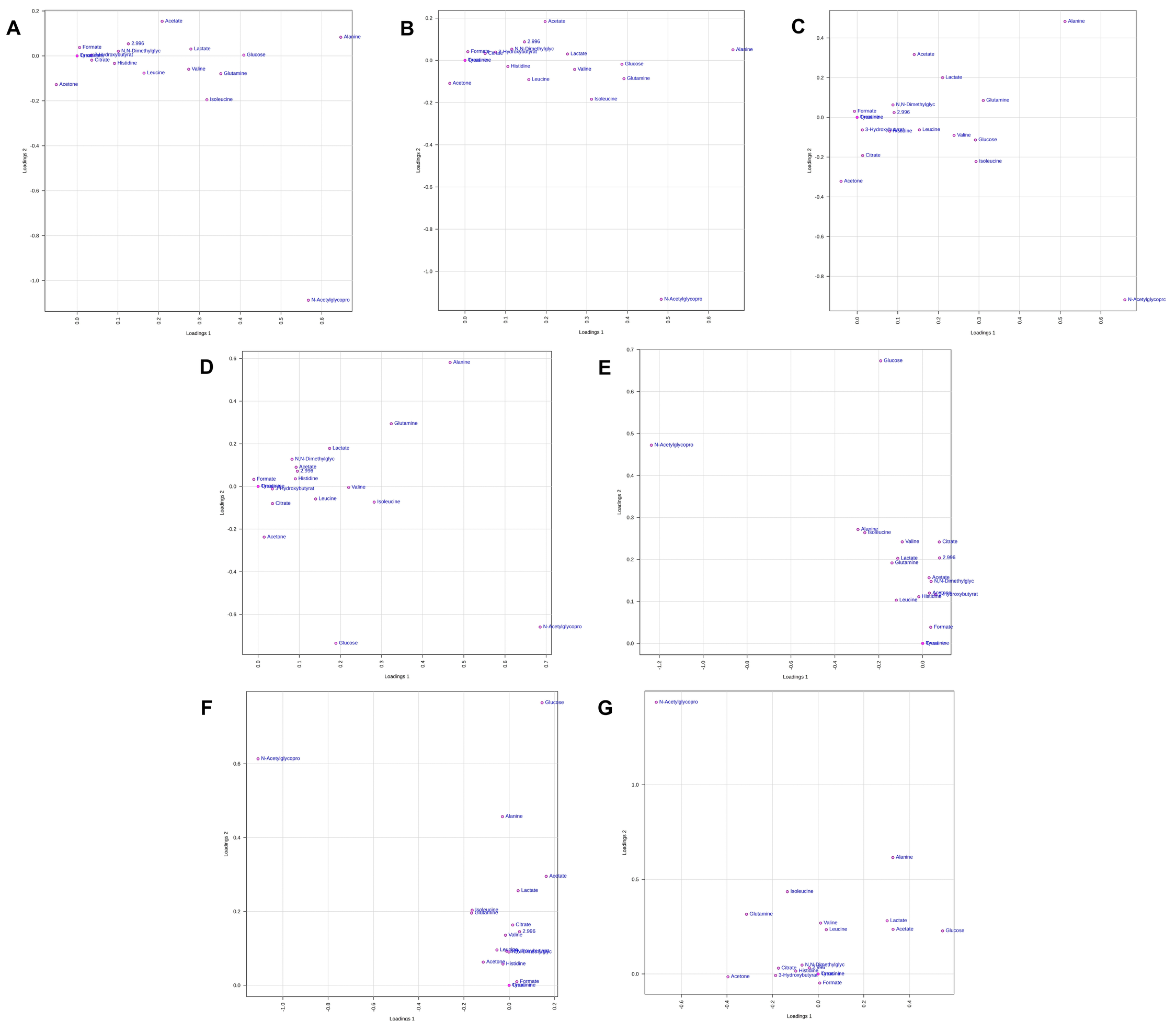

Supplement: S7 Fig — Loading plots of the PLS-DA comparing (A) healthy pregnant (HP) women, gestational hypertension subjects (GH), and preeclampsia patients without severe features (PE–) and preeclampsia patients with severe features (PE+), (B) HP and PE+, (C) GH and PE+ (D) PE–and PE+, (E) HP and GH, (F) HP and PE–, (G) GH and PE–. (TIF) [file pone.0314053.s007.tif]
