# Supplementary material for: Plasma metabolic profile reveals signatures of maternal health during gestational hypertension and preeclampsia without and with severe features
Source: PLoS One. 2024 Nov 26;19(11):e0314053. doi: 10.1371/journal.pone.0314053 (PMC11594399; doi:10.1371/journal.pone.0314053)
Supplement: Attachment — Submitted filename: Response to Reviewers.docx [file pone.0314053.s009.docx]

Journal: PLOS ONE | ISSN: 1932-6203

Manuscript ID: PONE-D-24-07221

**Paper:** “Plasma metabolic profile reveals signatures of maternal health during gestational hypertension and preeclampsia without and with severe features”

**Response to editor’s comments**

1. When submitting your revision, we need you to address these additional requirements.

Please ensure that your manuscript meets PLOS ONE's style requirements, including those for file naming. The PLOS ONE style templates can be found at

https://journals.plos.org/plosone/s/file?id=wjVg/PLOSOne_formatting_sample_main_body.pdf and

https://journals.plos.org/plosone/s/file?id=ba62/PLOSOne_formatting_sample_title_authors_affiliations.pdf

- We thank you for providing the additional requirements. The files were revised in accordance with the style requirements for file naming and the manuscript formatting guidelines set out by PLOS ONE.

2. Thank you for stating in your Funding Statement:

This work was supported by the Coordenação de Aperfeiçoamento de Pessoal de Nível Superior (CAPES) [grant number 88887.806462/2023-00 (JNSK)] , the Conselho Nacional de Desenvolvimento Científico e Tecnológico (CNPq) [grant numbers 308079/2021-3 (LT) and 308504/2021-6 (VCS)], and the Fundação de Amparo à Pesquisa do Estado de São Paulo (FAPESP) [grant numbers 2018/24069-3 (LT), 2019/07230-8, 2021/12010-7 (VCS), and 2023/08897-1 (JNSK)]. iBiMED is funded by Fundação para a Ciência e a Tecnologia (FCT) [UIDP/04501/2020 and UIDB/04501/2020 (MGA)]. The founders had no role in study design, data collection and analysis, decision to publish, or preparation of the manuscript.

Please provide an amended statement that declares all the funding or sources of support (whether external or internal to your organization) received during this study, as detailed online in our guide for authors at http://journals.plos.org/plosone/s/submit-now. Please also include the statement “There was no additional external funding received for this study.” in your updated Funding Statement.

Please include your amended Funding Statement within your cover letter. We will change the online submission form on your behalf.

- The Funding Statement was updated, and the following revised version was included in the cover letter:

“This work was supported by the Coordenação de Aperfeiçoamento de Pessoal de Nível Superior (CAPES) [grant number 88887.806462/2023-00 (JNSK)], the Conselho Nacional de Desenvolvimento Científico e Tecnológico (CNPq) [grant numbers 308079/2021-3 (LT) and 308504/2021-6 (VCS)], and the Fundação de Amparo à Pesquisa do Estado de São Paulo (FAPESP) [grant numbers 2018/24069-3 (LT), 2019/07230-8, 2021/12010-7 (VCS), and 2023/08897-1 (JNSK)]. iBiMED is funded by Fundação para a Ciência e a Tecnologia (FCT) [UIDP/04501/2020 and UIDB/04501/2020 (MGA)]. The founders had no role in study design, data collection and analysis, decision to publish, or preparation of the manuscript. There was no additional external funding received for this study.”

**Response to reviewers’ comments**

Please use the space provided to explain your answers to the questions above. You may also include additional comments for the author, including concerns about dual publication, research ethics, or publication ethics. (Please upload your review as an attachment if it exceeds 20,000 characters)

**Reviewer #1:** Review for Manuscript: “Plasma metabolic profile reveals signatures of maternal health during gestational hypertension and preeclampsia without and with severe features”.

This is an interesting manuscript on an important issue. The study design is retrospective study with 173 subjects. The authors have conducted a study on the metabolomic profile in maternal plasma during pregnancies affected by hypertensive disorders. The authors have used Nuclear magnetic resonance spectroscopy to determine the metabolomic profiles.
I have some recommendations for the authors:

1. The primary weakness of this study is its retrospective design, which may introduce biases and limitations that could affect the reliability of the findings. Authors should acknowledge and discuss these potential limitations in their manuscript.

- We would like to thank the reviewer for their constructive feedback. Upon reevaluation, we have identified that our study can be classified as a cross-sectional case-control design. We apologize for any confusion caused by the earlier misclassification. The necessary adjustments have been made in the Materials and Methods section (line 108). This type of study design has limitations, notably in establishing cause and effect relationships between metabolites and hypertensive disorders during pregnancy (lines 462-464).

2. The authors should explain why they have chosen nuclear magnetic resonance spectroscopy and give the advantages.

- This is a valuable suggestion, and we included a brief explanation in lines 84-89 (Introduction section).

3. A study flow chart may be helpful for the reader.

- We agree that a graphical representation of the study could enhance the reader's understanding and have now incorporated it into the study (Figure 1, lines 108 and 123). The final flow chart is presented below.

4. The authors have concluded that the exacerbated metabolic disturbance discloses renal impairment and hepatic dysfunction, evidenced by elevated levels of creatine and alanine. It would be better to use “may have disclosed”.

- We have carefully considered your feedback and have revised the passage accordingly to refine the clarity of our findings (line 40).

5. Body mass index of the healthy subjects is significantly lower than the other three groups. This may have caused a bias while comparing healthy with the other three groups. This should be stated.

- We agree that acknowledging potential biases is important in our analysis. It is worth noting that the BMIs were measured during pregnancy, a period characterized by physiological changes such as edema, particularly in cases of gestational hypertension and preeclampsia. It is possible that these fluctuations could influence BMI measurements. This aspect was highlighted in the Discussion section (lines 465-471) to evidence the potential impact on our comparisons.

6. The authors have collected the blood of preeclamptic women upon diagnosis. The study has a retrospective design. How did the authors get the blood samples?

- We extend our apologies once more for the misclassification of the study design. We reinforce that the samples were collected upon diagnosis and stored in a biobank (– 80 ºC). For the present study, we assessed the samples for conducting the RMN experiments (Material and methods section, line 151).

7. In the discussion section, the authors should address the clinical relevance of their findings. Specifically, they should discuss how their results may be applied in a clinical context.

- It is important to highlight the clinical relevance of our findings. We have incorporated this information into the Discussion section (lines 488-494). We appreciate your suggestions.

**Reviewer #2:** The health of expectant mothers and newborns can is seriously compromised by preeclampsia, a pregnancy-specific disease that can worsen over time. Studying the pathways and mechanisms underlying the pathophysiology of severe preeclampsia symptoms and the link with low molecular weight metabolites is an important issue. The topic is relevant because it refers to the health of the pregnant women. The manuscript has an excellent structure and description. The overall paper is organised and well-written. The tables are well-designed and comprehensible. The conclusions are consistent, and they address the main question. The references are appropriate.
I have some remarks:

The abbreviation of gestational hypertension is defined in Line 120 of the article. Before this, the two words are used more than once. I propose to define the abbreviation when you discuss gestational hypertension for the first time, and after that, it is recommended to use the abbreviation. In the tables, the abbreviations are defined many times. The same observation is made about the healthy pregnant abbreviation. In Line 458, Valine, an essential amino acid requiring dietary intake [20]. This reference does not discuss Valine, so I recommend checking it. I congratulate the authors for their efforts.

- We appreciate the reviewer for their valuable contributions to our study. Regarding the first point raised, we have amended and defined all the abbreviations upon their first usage, including within Table 1. Additionally, we believe it is preferable to retain the abbreviation definitions in the titles of images and table in order to ensure their standalone comprehensibility, allowing readers to interpret them independently of the main text. Furthermore, we have updated the valine reference in line 456 (PMID: 26950361), as well as in relevant instances (lines 384, 403, 426, 439).
