# Supplementary material for: Plasma metabolic profile reveals signatures of maternal health during gestational hypertension and preeclampsia without and with severe features
Source: PLoS One. 2024 Nov 26;19(11):e0314053. doi: 10.1371/journal.pone.0314053 (PMC11594399; doi:10.1371/journal.pone.0314053)
Supplement: Attachment — Submitted filename: Response to Reviewers.docx [file pone.0314053.s010.docx]

Journal: PLOS ONE | ISSN: 1932-6203

Manuscript ID: PONE-D-24-07221R1

**Paper:** “Plasma metabolic profile reveals signatures of maternal health during gestational hypertension and preeclampsia without and with severe features”

We thank the editor and all the reviewers for your valuable comments on our manuscript entitled “Plasma metabolic profile reveals signatures of maternal health during gestational hypertension and preeclampsia without and with severe features.” In the following section, we provide a point-by-point response to each comment, with the required revisions marked throughout the resubmitted manuscript ‘Revised Manuscript with Track Changes’.

**Response to editor’s comments**

Journal Requirements:

Please review your reference list to ensure that it is complete and correct. If you have cited papers that have been retracted, please include the rationale for doing so in the manuscript text, or remove these references and replace them with relevant current references. Any changes to the reference list should be mentioned in the rebuttal letter that accompanies your revised manuscript. If you need to cite a retracted article, indicate the article’s retracted status in the References list and also include a citation and full reference for the retraction notice.

We have thoroughly reviewed our reference list and can confirm that no retracted papers are included in this manuscript. We have ensured that all cited references are consistent and relevant to our study.

**Response to reviewers’ comments**

**Reviewer #3:** SUMMARY

In their manuscript, Kaihara et al report on an NMR based metabolomics study in plasma samples taken from pregnant women with the following pregnancy outcomes: Healthy Pregnancy (HP), Gestational Hypertension (GH), and preeclampsia with (PE+) and without severe features (PE-). For the non-HP women, samples were taken at time of diagnosis, whereas for the HP women, samples were obtained during routine clinical attendance. Samples were prospectively collected and biobanked prior to their analysis. The study design was a case-control study, with several case groups. The authors report differences between (normalised) the median metabolite levels across the different patient groups studied based on ANOVA analysis. In addition, they use PLA-DA and sPLS-DA to explore the ability of the metabolite profiles to differentiate patient groups and elicit the metabolites which contribute most to the class differentiations. In their reporting and interpretation, the authors first report on a global analysis comparing all patient groups and then focus on comparing / differentiating the PE(+) group from the others. In their discussion, the authors elaborate on extensively on the role of the key differentiating metabolites in their metabolite pathways to corroborate the plausibility of their results, where possible other metabolomics studies are referred to.

Overall, their manuscript is well written and well organised. In the main, the authors have performed and reported on a good piece of research and are commended for this work.

MAJOR ISSUES

Given that metabolite levels were not corrected for possible technical-, like gestation age at sampling, and patient confounders, e.g. BMI, age, smoking (not reported), maternal race/ethnicity (not reported), there is a non-negligent risk of over-interpretation of the results. Particularly, the authors focus a lot of their interpretation on the differences between the PE+ and other patient groups. Whereas it appears that metabolite levels in the PE+ are the most dysregulated as compared to the other patient groups, the gestational age of sampling is also on average 5 weeks earlier than in the other groups, which complicates interpretation (is it (mainly) a gestational age effect or is it a reflection of the presence of severe features?). In this context, the authors should consider refocusing their interpretation on differentiating HP from GH and/or PE- (association with hypertensive disorders of pregnancy/ differential association metabolite profiles between GH and PE). Given the syndromic nature of the hypertensive diseases and the spectrum in their presentation, the PE+ results could then be posited as an exacerbated dysregulation of the metabolite level (and its associated biochemical pathways). In the opinion of the reviewer, interpreting the data in this way sidesteps the gestational age of sampling critique and may add to the robustness of the data interpretation. For instance, the acetate levels (Figure 2) indicate that this metabolite is specific to PE, whereby its upregulated in PE- vs HP & GH; this is confirmed in the PE+, whereby the further upregulation is congruent with the assumption the PE+ is a worse form of PE. Similarly N,N-Dimethylglycine appears to associate with all hypertensive disorders of pregnancy (GH & PE), which its upregulation exacerbated in the PE(+).

We appreciate the reviewer’s valuable comments and suggestions regarding the interpretation of our results, particularly concerning the comparisons between PE+ and other patient groups. The emphasis on PE+ in our study was driven by the clinical significance and severity of this condition, with greater risks to both maternal and neonatal health, often necessitating earlier delivery as a clinical intervention. Therefore, the metabolite profile differences observed in PE+ are of particular interest, as they may provide insights into the pathophysiology of this disorder.

Regarding the gestational age at sampling, we acknowledge the potential confounding effect of gestational age at sampling, but it indeed tends to be earlier and is an inherent characteristic of the PE+ group. We analyzed correlations between metabolite levels and gestational age at sampling, finding that creatine, creatinine, and valine levels were negatively correlated, which could be a confounding factor in interpreting their associations with hypertensive disorders. This limitation has been included in our discussion. However, we believe that the metabolite dysregulation observed in the PE+ group is still of significant value, as no such correlations were found with other metabolites. Furthermore, we have incorporated the reviewer’s suggestions regarding the findings on acetate and *N*,*N*-dimethylglycine levels into the Discussion section. We believe that these revisions improved the quality of our manuscript.

MINOR ISSUES

ABSTRACT

1. Line 28: it is suggested to rephrase start of sentenced in line with study design as follows: Collected plasmas from…

2. Lines 29- 30: it is suggested to rephrase for readability: …preeclampsia without (PE-) and with (PE+) severe features

3. Lines 31-43: Depending on whether the authors heed the suggestion as per the Major Issue, this section may be rewritten.

We appreciate the reviewer’s suggestions for improving the abstract. We have incorporated the recommended changes to our manuscript.

INTRODUCTION

1. Line 72: it is suggested to rephrase for readability: Metabolites, vital components…

2. Line 85: it is suggested to rephrase for readability:… widely used approach for accurately quantifying…

3. Line 86: …Consider adding a reference to contemporary review re. use of 1H-NMR in metabolomics

4. Line 84: it is suggested to rephrase for readability: …. between placentas from pregnancies experiencing PE+ and from normotensive pregnancies.

5. Line 91: it is suggested to rephrase for readability: … to compare the metabolomic profiles ….

6. Lines 92-94: it is suggested to rephrase for readability: Studying the complex of changes… can offer…, assist in establishing … intricate landscape of hypertensive disorders of pregnancy.

7. Line 95: it is suggested to rephrase for readability:…mechanisms of the interrelated conditions GH, PE without and with severe features

8. Line 95-97: it is suggested to improve readability by removing sentence: However… or GH.

9. Lines 98-104: Depending on whether the authors heed the suggestion as per the Major Issue, this section may be rewritten.

We have incorporated the recommended changes to our manuscript, including a review regarding the use of ¹H-NMR in metabolomics (PMID: 31252628).

MATERIALS AND METHODS

1. Line 146: add time in pregnancy when blood of women with GH was collected

2. Line 147: it is suggested to rephrase for readability:… during routine clinical attendance

3. Line 151: NMR (instead of RMN)

We have incorporated the suggested changes into the Materials and Methods section of our manuscript.

RESULTS

1. Line 209: (Re-)Confirm BMI recorded in patient information is BMI at sampling. Typically, the BMI at begin of pregnancy or pre-pregnancy is considered. BMI at sampling may complicate data

2. Line 216 / TABLE 1: When plotting the creatinine levels as obtained by routine clinical testing vs the NMR creatinine read-outs, there is limited correlation (based on data provided in Supplementary Table 1). Can the authors explain this observation.

3. Lines 244: Inclusion of N-Acetylglycoproteins, clearly an aggregated non-metabolite compound may warrant some further discussion [DISCUSSION].

4. Lines 245- 259: Depending on whether the authors heed the suggestion as per the Major Issue, this section may be rewritten.

5. Lines 273-287: Depending on whether the authors heed the suggestion as per the Major Issue, this section may be rewritten / exchanged by e.g. HP vs GH and/or HP vs PE-, and interpretation of PE+ in these contexts (e.g. by plotting box plots HP vs GH vs PE+ and/or HP vs PE- vs PE+.

6. Line 282-287: elaboration on clustering as presented in Fig 3E. By including this clustering analysis, the authors indicate that there is some interesting insight to be gleaned from this clustering. However, the clustering results are not mentioned in the discussion? Based on the clustering presented, some commentary on the apparent subgroups in the PE+ class may be appropriate. Performing clustering within the classes PE+ and HP elicit these subgroups even more.

7. Lines 301-310: Depending on whether the authors heed the suggestion as per the Major Issue, this section may be rewritten. Highlighting differences between GH and PE+ has limited relevance when the metabolite levels are not different from HP in the first place.

8. Lines 323-331; equivalent comment as in Results - comment # 5

9. Lines 343-346: Description of S1 and S2 is better placed at start of results where metabolite identifications by NMR are reported.

10. Lines 346-353: Depending on whether the authors heed the suggestion as per the Major Issue, this section may be rewritten.

- In our study, BMI was recorded at the time of blood sampling, as pre-pregnancy measurements were not consistently available. As a result of the patients being referred from local health centers to the University Hospital of FMRP-USP, a tertiary care facility for high-risk pregnancies, data regarding their pre-pregnancy or early pregnancy BMI were unavailable for approximately 40% of the subjects. Additionally, it is possible that the pre-pregnancy BMI may not be a reliable indicator, due to the potential for memory biases to influence the recollection of pre-pregnancy weight information.
- In the PE+ group, concerning the correlation between creatinine levels obtained from routine clinical testing and those measured by NMR, we conducted a Spearman correlation test, given the non-parametric nature of the data. We observed a correlation between the two sets of creatinine levels (routine clinical testing on the y-axis and NMR on the x-axis), and the results are provided below (A). After excluding the evident outlier, the data distribution became parametric, and the correlation was sustained using the Pearson correlation test (B). We included these results in the manuscript (Metabolomics results section) and supplementary material (S3 Fig).

- We have now included a paragraph discussing the *N*-acetylglycoproteins findings.
- We agree that Fig 3E offers valuable insights into the PE+ group. To gain further understanding, we have conducted additional analyses using a hierarchical clustering algorithm with the metabolite concentration data within the PE+ group, followed by PLS-DA to represent the separation visually. This resulted in the formation of two distinct subgroups, designated as subgroups 1 (with 10 samples) and 2 (with 32 samples), as illustrated in S4 Fig. We generated a cluster dendrogram to identify the samples comprising each subgroup (S5 Fig). Subsequently, to explore potential contributing factors to the observed differences in metabolite concentrations within the PE+ group, we compared clinical, demographic, and biochemical data between the identified subgroups (S2 Table). We observed that while the subgroups are clinically quite similar, there is a trend towards differences in systolic and diastolic blood pressure, and proteinuria between subgroups 1 and 2. However, it is important to note that no international society dedicated to hypertension, obstetrics, or gynecology has established a classification system regarding these characteristics. Moreover, subgroup 1, which shows a trend of higher systolic and diastolic blood pressure, is composed of samples from patients who exhibited elevated levels of specific metabolites, especially leucine, valine, isoleucine, histidine, glutamine, alanine, and glucose as illustrated in the heatmap (Figure 3E).
- We have now moved the description of S1 and S2 Figs to the start of the results section.

DISCUSSION

1. Depending on whether the authors heed the suggestion as per the Major Issue, this section may be rewritten; writing itself is good. The authors should consider removing possible over-interpretative sections in the discussion, for instance (non-limiting examples):

a. lines 382-386: impact of glutamine on arginine bioavailability etc: health effects loosely attributed to downstream processes

b. Lines 386-389: higher glutamine levels (relative to glutamate; no data available) modulate risks/effects of hypertensive disorders. In their research, Kaihara et al observe an opposite association (again without glutamate data); citing contradictory research without explanation

c. Lines 412-421: impairment in renal function is a hallmark of PE, cf. proteinurea is one of the diagnostic criteria; the reviewer would be more interested in the apparent lack of association between NMR creatinine and clinical chemistry creatinine

d. Lines 422-437: Alanine levels may reflect differences in AA trajectories (https://doi.org/10.3390/nu13093080) in pregnancy; possibly associated with dynamically changing metabolic needs as per the authors discussion

We have reviewed the mentioned sections regarding glutamine and revised the discussion to focus more directly on the findings of our study, emphasizing that while the connections between glutamine levels and the disorder are plausible, they require further research for confirmation. To avoid potential confusion, we also removed the information on the relative levels of glutamine and glutamate, and instead only refer to the results of glutamine levels. Furthermore, we have removed the reference to the genetic deletion of glutamine synthetase. As addressed earlier, we conducted Spearman and Pearson (when excluding the outlier) correlations tests and there is indeed an association between NMR creatinine and clinical testing creatinine levels, which we have included in the manuscript. Additionally, we have included a reference regarding alanine levels during pregnancy.

CONCLUSIONS

1. Depending on whether the authors heed the suggestion as per the Major Issue, this section may be rewritten.

We appreciate the reviewer's suggestions and comprehend the rationale behind them. However, the manuscript has already been subjected to a thorough review process and has been accepted by two previous reviewers with a specific focus on PE+. A change in the main focus of the article at this point could result in a notable shift in the direction and emphasis of the research, which could conflict with previously accepted findings from peer reviewers. Then, as the major revisions were not incorporated, the conclusion section remains unchanged.

**Reviewer #4:** The study by Julyane N. S. Kaihara and co-authors provides an excellent analysis of metabolic profiles of preeclampsia women compared with both gestational hypertension and healthy individuals. Moreover, PE women were divided into subgroups according to the severity of symptoms. The paper is accessible, and conclusions are given based on the results. The scientific background of the study is vivid, and the presented data shed light on the pathogenesis of preeclampsia and gestational hypertension.

The given version of the paper was revised after the previous review round. The authors' answers were sufficient, and the text was improved accordingly.

I have only minor suggestions for the authors.

1. The paper would benefit from the correlation analysis of metabolomics results with the clinical characteristics of the study populations.

We agree with this suggestion and have incorporated correlation analyses between the metabolomics results and the clinical characteristics of the study cohort.

2. The patient’s characteristics should be moved from the result section to the material and method section.

We appreciate this reviewer’s suggestion; however, we believe that presenting these characteristics at the beginning of the Results section is more appropriate for this study. This allows for an understanding of the clinical and demographic characteristics of the study population before the metabolomics results. We believe that this structure enhances the clarity and coherence of the manuscript.
