# Supplementary material for: Plasma metabolic profile reveals signatures of maternal health during gestational hypertension and preeclampsia without and with severe features
Source: PLoS One. 2024 Nov 26;19(11):e0314053. doi: 10.1371/journal.pone.0314053 (PMC11594399; doi:10.1371/journal.pone.0314053)
Supplement: Attachment — Submitted filename: Response to Reviewers.docx [file pone.0314053.s011.docx]

Journal: PLOS ONE | ISSN: 1932-6203

Manuscript ID: PONE-D-24-07221R2

**Paper:** “Plasma metabolic profile reveals signatures of maternal health during gestational hypertension and preeclampsia without and with severe features”

We thank the editor and all the reviewers for your valuable comments on our manuscript entitled “Plasma metabolic profile reveals signatures of maternal health during gestational hypertension and preeclampsia without and with severe features.” In the following section, we respond to each comment, with the required revisions marked throughout the resubmitted manuscript ‘Revised Manuscript with Track Changes’.

**Response to editor’s comments**

Journal Requirements:

Please review your reference list to ensure that it is complete and correct. If you have cited papers that have been retracted, please include the rationale for doing so in the manuscript text, or remove these references and replace them with relevant current references. Any changes to the reference list should be mentioned in the rebuttal letter that accompanies your revised manuscript. If you need to cite a retracted article, indicate the article’s retracted status in the References list and also include a citation and full reference for the retraction notice.

We have thoroughly reviewed our reference list and can confirm that no retracted papers are included in this manuscript. We have ensured that all cited references are consistent and relevant to our study.

**Response to reviewers’ comments**

**Reviewer #3:** SUMMARY

In their manuscript, Kaihara et al report on an NMR based metabolomics study in plasma samples taken from pregnant women with the following pregnancy outcomes: Healthy Pregnancy (HP), Gestational Hypertension (GH), and preeclampsia with (PE+) and without severe features (PE-). For the non-HP women, samples were taken at time of diagnosis, whereas for the HP women, samples were obtained during routine clinical attendance. Samples were prospectively collected and biobanked prior to their analysis. The study design was a case-control study, with several case groups. The authors report differences between the (normalised) median metabolite levels across the different patient groups studied based on ANOVA analysis and pairwise group analyses. In addition, they use PLA-DA and sPLS-DA to explore the ability of the metabolite profiles to differentiate patient groups and elicit the metabolites contributing most to the class differentiations. In their reporting and interpretation, the authors first report on a global analysis comparing all patient groups and then focus on comparing / differentiating the PE(+) group from the others. In their discussion, the authors elaborate on extensively on the role of the key differentiating metabolites in their metabolite pathways to corroborate the plausibility of their results, where possible other metabolomics studies are referred to.
Overall, upon revision, their manuscript remains well written and well organised. In the main, the authors have performed and reported on a good piece of research.

MAJOR ISSUES

None

MINOR ISSUES

Notwithstanding, the good writing and logical organisation of the manuscript, the reviewer regrets the authors didn’t take the opportunity to reorganize their manuscript and to either posit PE(+) to be on a HP > GH > PE(-) > PE(+) spectrum. By adopting a PE(+) centric view on the study, the authors missed the opportunity discussing whether metabolites can differentiate HP from GH and/or PE and/or PE(+). Having all these subgroups represented in one of the strong appeals of this research. As a result of the authors choice, the manuscript lacks a clear narrative and clear conclusions. It remains unclear what metabolites are adding true value in the understanding of the hypertensive diseases of pregnancy. This is obvious in the metabolites the authors chose to elaborate upon in the discussion.

The reviewer had hoped that the authors would considerably prune their manuscript based on the feedback as provided on the previous version, unfortunately this advice was not heeded. For instance, the authors added a lot of results with regards to the presence of sub-clusters in the PE(+) group. Whereas this work was triggered by the reviewer (and is potentially informative), the clustering and extra results are not featuring in the discussion. If results are not informative in the context of the study (cf. they don’t feature in the discussion), it is better to remove the clustering analysis altogether rather than adding more to it. Same remains true for the contextualisation of the metabolites in the discussion, the contextualisation remains unwieldy leaving the reader with little additional insight as a result of the study.

In summary, the researchers did a good piece of research, but -in the reviewer’s opinion- remained to keen to put too much in the paper making the dissemination of their research less effective than it could be.

Thank you for your constructive and helpful feedback. Our primary focus on the PE+ group, as previously stated, was driven by its significant impact on maternal health and the severity of its clinical consequences, as well as the importance in maintaining a cohesive and concise manuscript. We agree that the incorporation of the heatmap and the PE+ subgroup analysis slightly deviated from this focus, and then we have removed these analyses from the manuscript. Furthermore, we chose to keep the spotlight on PE+ while making minor amendments throughout the text to improve its overall clarity and quality. We hope the revised version is more suitable.

ABSTRACT
1. OK

INTRODUCTION
1. Line 52: replace “necessitates” by “often necessitates”

2. Line 61: replace "premature termination of the pregnancy” by “iatrogenic premature delivery” to avoid confusion with abortion

3. Line 70: replace “as accompanied by” with “together with”
Lines 71-72: replace "the intricate landscape of” by “the complexity of”

Line 96-101: too verbose, simplify by 1) removal of “Studying…pregnancy” 2) Add “Studying the levels of metabolites across the different hypertensive disorders of pregnancy may provide deeper insights into…” 3) remove “it underscores the….PE+. Thus” 4) Restart: In this study our primary…

4. Line 107: Rephrase” Subsequently, we investigated the presence of correlations…

MATERIALS AND METHODS

1. Line 191: Add : “ For pairwise group comparisons, post-hoc tests…. “

2. Lines 211-217: Suggestion to temove clustering section

RESULTS
1. Suggestion to remove lines 310 – 315: “Moreover,… clusters”

2. Suggestion in Fig 3: remove panel E

3. Suggestion to remove Description of Figure 3: panel E, lines 325 -327, remove all affiliated supplementary results.

4. Suggestion to Remove lines 328-346

DISCUSSION
1. OK

CONCLUSIONS
1. OK

We have incorporated these suggestions into our manuscript. To maintain the focus on PE+, the heatmap and clustering analyses related to the subgroups have been removed.

**Reviewer #4:** All the questions given to the authors has been adressed accordingly. I have no more questions.

We appreciate the reviewer's valuable suggestions and contribution to improving our paper.
